# Sitagliptin Attenuates the Cognitive Deficits in L-Methionine-Induced Vascular Dementia in Rats

**DOI:** 10.1155/2022/7222590

**Published:** 2022-02-27

**Authors:** Suzan A. Khodir, Manar A. Faried, Huda I. Abd-Elhafiz, Eman M. Sweed

**Affiliations:** ^1^Medical Physiology Department, Faculty of Medicine, Menoufia University, Menoufia 32511, Egypt; ^2^Human Anatomy and Embryology, Faculty of Medicine, Menoufia University, Menoufia 32511, Egypt; ^3^Clinical Pharmacology Department, Faculty of Medicine, Menoufia University, Menoufia 32511, Egypt

## Abstract

Vascular dementia (VaD) is the second most prevalent type of dementia characterized by progressive cognitive deficits and is a major risk factor for the development of Alzheimer's disease and other neurodegenerative disorders. This study is aimed at determining the potential neuroprotective effect of sitagliptin (STG) on cognitive deficits in L-methionine-induced VaD in rats and the possible underlying mechanisms. 30 adult male Wistar albino rats were divided equally (*n* = 10) into three groups: control, VaD, and VaD + STG groups. The cognitive performance of the animals was conducted by open field, elevated plus maze, Y-maze, novel object recognition, and Morris water maze tests. Serum homocysteine, TNF-*α*, IL-6, IL-10, total cholesterol, and triglycerides levels were assessed together with hippocampal MDA, SOD, and BDNF. Histopathological and immunohistochemical assessments of the thoracic aorta and hippocampus (CA1 region) were also performed. Chronic L-methionine administration impaired memory and learning and induced anxiety. On the other hand, STG protected against cognitive deficits through improving oxidative stress biomarkers, inflammatory mediators, lipid profiles, and hippocampus level of BDNF as well as decreasing caspase-3 and GFAP and increasing Ki-67 immunoreactions in the hippocampus. Also, STG improved the endothelial dysfunction via upregulation of aortic eNOS immunoreaction. STG improved the cognitive deficits of L-methionine-induced VaD by its antioxidant, anti-inflammatory, antiapoptotic, and neurotrophic effects. These findings suggest that STG may be a promising future agent for protection against VaD.

## 1. Introduction

Vascular dementia (VaD) is a neurodegenerative disease characterized by progressive cognitive deficits with a negative impact on patient's quality of life. It is considered the second most prevalent type of dementia worldwide, resulting in extensive medical and economic troubles in society [1].

Vascular dementia (VaD) is caused by cumulative hippocampal neuronal damage caused by vascular factors [2]. Several risk factors are associated with VaD, including cardiovascular abnormalities, dyslipidaemia, obesity, smoking, diabetes, and increased homocysteine (Hcy) level [3]; targeting these risk factors will minimize the burden. One of the major risk factors for VaD is hyperhomocysteinemia (HHcy) [4]. Also, some survey results showed that Hcy levels were significantly elevated in patients with anxiety [5].

Methionine is an essential amino acid obtained from food, as it cannot be synthesized endogenously. The amino acid Hcy is a significant by-product of methionine metabolism [6]. Hcy accumulation could disrupt endothelial function, cause oxidative damage, and increase neuroinflammatory and neurodegenerative processes [7]. As a result, oxidative stress, inflammation, and neuronal apoptosis have all been linked to the pathophysiology of VaD [8]. Hcy neurotoxicity is hypothesized to occur predominantly through increased oxidative stress in the brain, as well as decreased nitric oxide bioavailability, resulting in endothelial dysfunction, decreased cerebral blood flow, and, eventually, memory impairment [9].

Recent evidence indicates that hippocampal neurogenesis perpetuates throughout life [10]. Hippocampal neurogenesis was downregulated in VaD with the subsequent deterioration of the cognitive function. In the adult brain, brain-derived neurotrophic factor (BDNF) is known to be important in synaptic plasticity, learning, and neurogenesis [11]. BDNF has been identified to regulate neuronal survival and influence cognitive processes [12]. Moreover, Ki-67 is an effective mitotic marker gauging the proliferation in the initial phase of adult neurogenesis [13].

Due to the extensive focus on pharmacological intervention to alleviate neurodegenerative changes in VaD, devising a drug that has antioxidant and anti-inflammatory properties and promotes hippocampal neurogenesis is of a great impact.

Dipeptidyl peptidase-4 (DPP-4) inhibitors, including sitagliptin (STG), are among the drugs studied for their potential use for new therapeutic purposes as a part of a drug repositioning or repurposing strategy [14]. STG is effectively used in the treatment of type 2 diabetes mellitus [15]. Moreover, DPP-4 inhibitors exhibit neuroprotective effects against neuronal degeneration [16]. STG augments the circulating glucagon-like peptide-1 (GLP-1) levels via inhibition of DPP-4 activity. Accordingly, GLP-1 might have a neuroprotective role via its beneficial effects on brain physiology and learning behavior [17].

To the best of our knowledge, the potential role of STG in methionine-induced VaD has not been evaluated, although it is an interesting candidate as free radicals play a major role in the pathogenesis of dementia while antioxidants play an important role in the alleviation of dementia. STG has been reported as a potential antioxidant [18]. Consequently, this study was designed to investigate, for the first time up to our knowledge, the antioxidant, anti-inflammatory, antiapoptotic, and neurotrophic roles of STG in L-methionine-induced VaD.

## 2. Materials and Methods

### 2.1. Animals

This study was conducted on 30 adult (three months old, weighing 200 ± 50 g) male Wistar albino rats. They were maintained in the animal house of the Faculty of Medicine, Menoufia University, Egypt, under standard conditions with a natural light–dark cycle. They were fed with standard rat chow and had free access to water. Rats were left to acclimatize for a week before the experiment.

### 2.2. Ethical Statement

All procedures were conducted per the Committee of Animal Research Ethics, Menoufia University's Faculty of Medicine's guidelines under IRB number (9/2021PHAR3-B). The ARRIVE reporting guidelines were followed.

### 2.3. Experimental Design

Sample size was calculated according to the study design and the objectives of the study. This study is an experimental study. The main objective is to investigate the potential protective effect of STG in L-methionine-induced VaD in rats. Guided by the previous literature, and at CI of 95% and study power of 80% (0.8), the sample size was 30. Rats were randomly divided into three equal groups (10 rats each):

Group I (control): the rats were kept without treatment until the end of the experiment.

Group II (L-methionine-induced VaD group) [VaD]: L-methionine (purchased from Sigma, St Louis, MO, USA, in the form of powder) was administered at a dose of 1700 mg/kg, dissolved in distilled water, and given once daily by oral gavage for 32 days to induce VaD [19].

Group III (L-methionine-induced VaD + STG treated group) [VaD + STG]: starting from the first day of L-methionine administration (at the same dose, duration and route of administration as group II), the rats received also STG at a dose of 10 mg/kg, dissolved in distilled water once daily by oral gavage [20, 21] for 32 days. STG (JANUVIA 100 mg) was purchased from Merck Sharp and Dohme Corp. (a subsidiary of Merck and Co., Inc., Kenilworth, NJ, USA).

The cognitive performance of the animals was assessed during the last five days of the experiment. After the behavioral tests, blood samples were collected from the retro-orbital venous plexus for biochemical assessment, and the rats were sacrificed by cervical dislocation under anaesthesia (intraperitoneal injection of 60 mg/kg phenobarbital). Thoracic aorta and cerebral hemispheres were dissected for the assessment of vascular affection and neurodegenerative changes, respectively. The right hippocampi were dissected and immediately frozen at a temperature of -80°C for further biochemical study. The left cerebral hemispheres were processed for the histological and immunohistochemical study of the hippocampus (CA1 region).

### 2.4. Cognitive Performance Assessment

#### 2.4.1. Open Field Test

A wooden arena was divided into 25 squares (100 × 100 × 60 cm height, black wall and floor) (20 cm per square). For 15 minutes, a rat was placed in the center of the arena and permitted to freely explore it [22]. The time spent in the central zone was measured, and the total distance moved in meters (m) was calculated by counting the number of crossed squares.

#### 2.4.2. Elevated Plus Maze (EPM) Test

Anxiety-like behavior in rats was measured using a plus-sign shape device, as previously described [23]. Briefly, rats were placed separately in device's central region and permitted to explore the maze for ten minutes. An overhead camera was used to track the movements of the animals. The time spent in the open arms of the maze was recorded. The time an animal spent in the open arms was inversely proportional to its anxiety-like behavior.

#### 2.4.3. Y-Maze Test

The Y-maze test is used in rodents to assess spatial working memory [24]. For eight minutes, each rat was placed at one end of the arm and permitted to freely navigate through the maze. An arm entry was recorded when all four paws of the rats were within any of three arms. Alterations were defined as entries into all three arms on successive options, i.e., ABC, CAB, or BCA—but not BAB. A Sony video camera was used to capture the series of arm entry, including possible returns into the same arm. Calculation of the percentage of alternation was calculated using the following formula:

(1) $$\begin{array}{c} \%\text{of alternation}=\frac{\left(\text{Total number of alternation}\right)}{\left(\text{Total number of arm entries}-2\right)}\times 100. \end{array}$$

#### 2.4.4. Novel Object Recognition (NOR) Test

During the last three days of the trial, rats' cognitive function and memory were assessed using the NOR test [25]. A wooden open rectangular box was utilized (65 × 45 × 65 cm).

In the NOR test, two distinct opaque cubes (familiar objects) and a blue ball (novel object) with an opaque cube were utilized. The NOR test was divided into three phases: habituation, familiarization, and testing. During the habituation phase, rats were individually placed in the box and given three minutes to explore the empty open field arena. Two trials (T1 and T2) were conducted 20 hours following the habituation trial, separated by an intertrail interval (24 hours). Rats were placed in the open field during T1 (familiarization phase) and allowed to explore two identical familiar objects (a1 and a2).

Following the intertrial interval, the T2 (test phase) was carried out with a novel object (b) and a new familiar object (a). A stopwatch was used to record the time the rats spent exploring the objects during T1 and T2, and live videos were filmed to study rats' activities. Rats were regarded as exploring when they directed their noses 2 cm from an object or touched it. The test lasted three minutes, and the contact time with the items (20 seconds) was fixed in T1 and T2 to ensure test's sensitivity and comparability. The total time rats spent exploring similar objects in the familiarization phase (T1) was calculated.

Furthermore, the recognition index (RI) was determined, which is the time spent investigating the novel object compared to the total time spent investigating the novel and familiar objects. To examine memory performance, one familiar object was replaced with a novel object during the testing session.

#### 2.4.5. Morris Water Maze (MWM) Test

During the final five days of the experiment, the Morris water maze (MWM) test was performed. MWM's circular pool was partitioned into four equal quadrants. For the first four days, a platform (diameter 10 cm) was placed 1 cm below the surface of the water in one of the quadrants. Each rat was placed in one of the three randomly selected locations in the pool on each day of the acquisition session (three trials each session).

The trial was started by immersing the rat in the pool. The trial was ended when the rat discovered and climbed onto the platform, and the mean escape latency was calculated. The maximum duration of the test was 60 seconds. If the rat did not climb up the platform within 60 seconds, it was gently placed on it, and the time was recorded as 60 seconds.

On the fifth day, a “probe trial” was conducted to measure the rat's remembering of the position of the hidden platform within 60 seconds. During this trial, the platform was removed from the pool [26].

### 2.5. Blood Sampling and Biochemical Analysis

At the end of the experiment, animals did not feed overnight, and then, blood samples were collected, allowed to coagulate for 30 minutes at room temperature. Then, they were centrifuged at 2000 rpm for 15 minutes. The serum was collected and frozen at a temperature of -80°C until the time of analysis. Serum Hcy, tumor necrosis factor-alpha (TNF-*α*), interleukin 6 (IL-6), and interleukin 10 (IL-10) levels were measured using the corresponding rat enzyme-linked immunosorbent assay (ELISA) kits (Hcy: 201-11-1646, Shanghai Sunred Biological Technology Co., Ltd, China, TNF-*α*: ERT2010-1, Assaypro LLC, Saint Charles, Missouri, USA, IL-6: ab100772, Abcam, Cambridge, UK, and IL-10: ERI3010-1, Assaypro LLC, Saint Charles, Missouri, USA according to manufacturer's instructions). Fasting serum lipids (total cholesterol and triglyceride) were determined using colorimetric kits (Biodiagnostic Company, Dokki, Giza, Egypt).

### 2.6. Tissue Homogenate Preparation

Weighted hippocampus specimens were homogenized separately with a tissue homogenizer (MPW120, MPW Medical Instruments, China). Calorimetric kits were used to measure hippocampus malonaldehyde (MDA) and superoxide dismutase (SOD) (Biodiagnostic Company, Dokki, Giza, Egypt). The BDNF in hippocampus was measured using the matching rat ELISA kits (Kit Catalogue Number: SL0131Ra, SunLong Biotech Co., LTD, Hangzhou, China) in accordance with manufacturer's instructions.

For the analysis of MDA and BDNF tissues, they were homogenized in a phosphate buffer saline (PBS) 50 mM pH 7.4. For the analysis of SOD, tissues were homogenized in a potassium phosphate buffer (PPB) 10 mM pH 7.4. The crude tissue homogenate was centrifuged at 10000 rpm for 15 minutes in an ice-cold centrifuge, and the resultant supernatant was collected and stored at a temperature of −80°C for assay.

### 2.7. Histological and Immunohistochemical Evaluation

For histopathological assessment, the specimens from the thoracic aorta and the left cerebral hemisphere were preserved in 10% neutral formaldehyde and processed for light microscopic examination. For routine histological investigation, paraffin slices (5 *μ*m thick) were produced and stained with haematoxylin and eosin (H&E).

The deparaffinized and rehydrated 5 *μ*m slices were washed with PBS and blocked in a 3% H_2_O_2_ compound as an inhibitor of endogenous peroxidase activity for the immunohistochemical study. The microwave antigen retrieval method was carried out after rinsing in PBS.

*Assessment of Endothelial Dysfunction:* The sections from the thoracic aorta were incubated with the primary antibody: endothelial nitric oxide synthase (eNOS) [1 : 1000, mouse monoclonal, Abcam ab76198].

*Assessment of Hippocampal Degeneration:* The sections from the brain were incubated with the primary antibodies: caspase-3 [1 : 1000, rabbit monoclonal, Abcam ab184787], glial fibrillary acidic protein (GFAP) [1 : 300, mouse monoclonal, Lab vision MS-1376-R7], and Ki-67 [1 : 200, rabbit monoclonal, Abcam ab16667].

The sections were incubated an hour at room temperature with the various primary antibodies. The sections were then washed with PBS before being incubated for 20 minutes at room temperature with a secondary biotinylated antibody. After washing the sections in PBS, a ten-minute application of an enzyme conjugate “Streptavidin Horseradish peroxidase” solution was provided to the sections. 3,3-Diaminobenzoic acid (DAB)was used to visualize the secondary antibody binding. Finally, the sections were washed in PBS, and the slides were counterstained with two drops of haematoxylin.

### 2.8. Morphometric Study

For morphometric assessment, nonoverlapping fields (×400) per section in three different serial sections from each rat were obtained using a Leica DML B2/11888111 microscope equipped with a Leica DFC450 camera. The examined parameters were calculated using ImageJ software version K1.45.

#### 2.8.1. Aortic Morphometric Assessment

From H&E–stained sections, the intima-media thickness (IMT) was measured: four measurements of IMT per section were obtained at 0°, 90°, 180°, and 270°. The measurements acquired were averaged to get the value corresponding to the single section [27]. Finally, the IMT for each animal was estimated by calculating the average value of the three measured aortic sections [28].

For immunohistochemical quantitative assessment, five nonoverlapping fields/sections were assessed for the percentage of eNOS immunopositive endothelial cells.

#### 2.8.2. Hippocampal Morphometric Assessment

From H&E-stained sections, the percentage of degenerated neurons (number of degenerated cells/total cells × 100) was recorded in three nonoverlapping fields/section.

For immunohistochemical quantitative assessment, the percentage of caspase-3 and Ki-67 immunopositive cells and the area percentage of GFAP immunoreaction were measured in three nonoverlapping fields/sections.

### 2.9. Statistical Analysis

The SPSS version 23 (SPSS, Inc., USA) was used for the analysis of data. Shapiro-Wilk test was performed on all data sets to ensure normal distribution. The results were expressed as mean ± standard deviation (SD). The significance of differences between groups was determined by one-way analysis of variance (ANOVA) followed by a post hoc Tukey test. *P* values less than 0.05 were considered statistically significant.

## 3. Results

### 3.1. The Effect of STG on Serum Hcy Level in L-Methionine-Induced VaD

**Figure 1:**
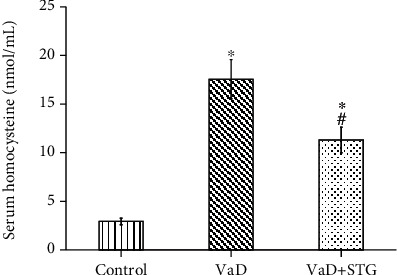
Effect of STG on serum homocysteine level in L-methionine-induced VaD (VaD: vascular dementia; VaD+STG: vascular dementia+sitagliptin). Each group (*n* = 10) represents as mean ± SD. ^∗^*P* < 0.001 versus the control group; ^#^*P* < 0.001 versus the VaD group.

The mean value of serum Hcy level in the VaD group was significantly higher than that of the control group (17.55 ± 2.02 vs. 2.93 ± 0.34 nmol/mL, respectively, *P* < 0.001). Serum Hcy level in the VaD + STG group was significantly lower than that of the VaD group's (11.3 ± 1.31 nmol/mL, *P* < 0.001); however, it was still significantly higher than the control group's (*P* < 0.001) (see Figure 1).

### 3.2. The Effects of STG on Cognitive Performance in L-Methionine-Induced VaD

#### 3.2.1. Open Field Test

**Figure 2:**
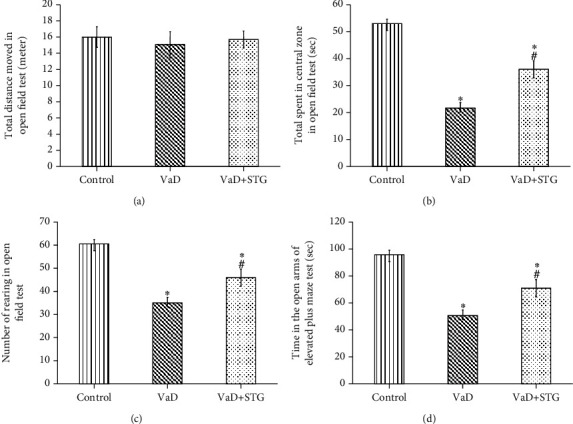
Effect of STG on open field and elevated plus maze tests parameters in L-methionine-induced VaD (VaD: vascular dementia; VaD+STG: vascular dementia+sitagliptin). Each group (*n* = 10) represents as mean ± SD. (a) Total distance moved in open field test (meters). (b) Time spent in central zone (seconds). (c) The number of rearing. (d) Time in the open arms of elevated plus maze test. ^∗^*P* < 0.001 versus the control group; ^#^*P* < 0.001 versus the VaD group.

The total distance moved in the open field test in the VaD group showed no statistically significant difference when compared with the control group's values (15.12 ± 1.67 vs. 16 ± 1.44 meters, respectively, *P* > 0.05). The total distance moved in the open field test in the VaD+STG group showed no statistically significant difference when compared with the values of the VaD and control groups (15.53 ± 1.16 meter, *P* > 0.05) (see Figure 2(a)).

The time spent in the central zone in the open field test in the VaD group was significantly lower than that of the control group values (21 ± 1.89 vs. 51.3 ± 2.25 seconds, respectively, *P* < 0.001). The time spent in the central zone in the open field test in the VaD+STG group was significantly higher than that of the VaD group (36.17 ± 2.13 seconds, *P* < 0.001), even though it was still significantly lower than that of the control group (*P* < 0.001) (see Figure 2(b)).

The number of rearing in the open field test in the VaD group was significantly lower than that of the control group (34.5 ± 1.64 vs. 59.5 ± 2.26, respectively, *P* < 0.001). The number of rearing in the open field test in the VaD+STG group was significantly higher than that of the VaD group (45.5 ± 1.62, *P* < 0.001), yet it was still significantly lower than the control group's (*P* < 0.001) (see Figure 2(c)).

#### 3.2.2. The EPM Test

There was a significant decrease in the time spent in the open arms of the EPM test in the VaD group compared with the control group's (48.67 ± 3.14 vs. 94.33 ± 3.2 seconds, respectively, *P* < 0.001). The time in the open arms of the EPM test level in the VaD + STG group was significantly higher than that of the VaD group's (69.17 ± 3.37 seconds, *P* < 0.001); however, it was still significantly lower than the control group values (*P* < 0.001) (see Figure 2(d)).

#### 3.2.3. Y-Maze Test

**Figure 3:**
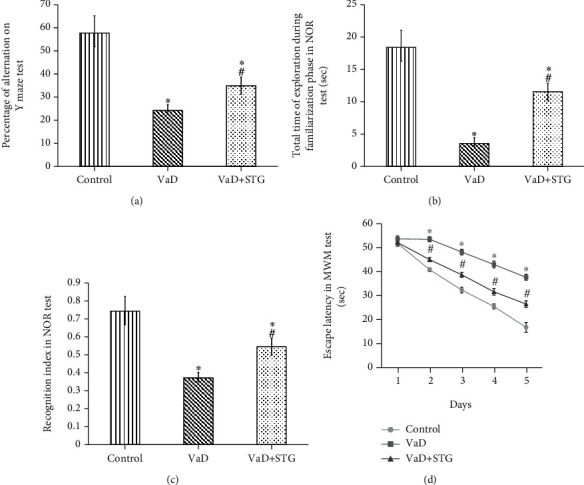
Effect of STG on Y-maze, NOR, and MWM test parameters in L-methionine-induced VaD (VaD: vascular dementia; VaD+STG: vascular dementia +sitagliptin; NOR: novel object recognition; MWM: Morris water maze). Each group (*n* = 10) represents as mean ± SD. (a) The percentage of alternation on Y-maze test. (b) The total time of exploration during familiarization phase in NOR. (c) The recognition index in NOR. (d) The escape latency in the 2nd, 3rd, 4th, and 5th days of MWM test. ^∗^*P* < 0.001 versus the control group; ^#^*P* < 0.001 versus the VaD group.

The percentage of alternation during the Y-maze test in the VaD group was significantly lower than that of the control group (22.18 ± 3.09 vs. 57.98 ± 7.17, respectively, *P* < 0.001). There was a significant increase in the percentage of alternation during the Y-maze test levels in the VaD+STG group compared with that of the VaD group (33.58 ± 2.23, *P* < 0.001), even though it was still significantly lower than that of the control group (*P* < 0.001) (see Figure 3(a)).

#### 3.2.4. The NOR Test

The total time of exploration during the familiarization phase in the NOR level in the VaD-group was significantly lower than that of the control group (3.83 ± 0.7 vs. 19.166 ± 1.94 seconds, respectively, *P* < 0.001). On the other hand, the total time of exploration during the familiarization phase in the NOR level in the VaD+STG group was significantly higher than that of the VaD group (11 ± 1.67 seconds, *P* < 0.001), yet it was still significantly lower than that of the control group (*P* < 0.001) (see Figure 3(b)).

There was a significant decrease in the RI in the NOR in the VaD-group compared with that of the control group (0.37 ± 0.04 vs. 0.75 ± 0.05, respectively, *P* < 0.001). The RI in the NOR level in the VaD+STG was significantly higher than in the VaD group (0.523 ± 0.03, *P* < 0.001); however, it was still significantly lower than the control group values (*P* < 0.001) (see Figure 3(c)).

#### 3.2.5. The MWM Test

The mean value of the escape latency on the first day of the MWM test in the VaD group showed no statistically significant difference when compared with that of the control group (53.66 ± 1.21 vs. 51.8 ± 1.16 seconds, respectively, *P* > 0.05). The escape latency in the first day of the MWM test in the VaD+STG showed no statistically significant difference when compared with the VaD and control groups (52.16 ± 1.47 seconds, *P* > 0.05).

The mean value of the escape latency on the second, third, fourth, and fifth days of the MWM test in the VaD-group was significantly higher than those of the control group (53.5 ± 1.04, 48.17 ± 1.16, 43 ± 1.41, 37.66 ± 1.21 vs. 40.8 ± 0.75, 32.33 ± 1.21, 25.5 ± 1.04, 16.8 ± 2.04 seconds, respectively, *P* < 0.001). The escape latency in the second, third, fourth, and fifth days of the MWM test level in the VaD+STG was significantly lower than those of the VaD group (45 ± 0.89, 38.66 ± 1.03, 31.6 ± 1.37, and 26.5 ± 1.37 seconds, respectively, *P* < 0.001). On the other hand, the values were still significantly higher than those of the control group (*P* < 0.001) (see Figure 3(d)).

There was a significant difference between the different groups regarding the percentage of change of latency between day 1 and day 5 in MWM test (*P* < 0.001). VaD+STG group revealed a significant improvement in the percentage of change of latency between day 1 and day 5 in MWM test when compared to the VaD group (*P* < 0.001) (Table 1).

### 3.3. The Effects of STG on Oxidative Stress Biomarkers in L-Methionine-Induced VaD

**Figure 4:**
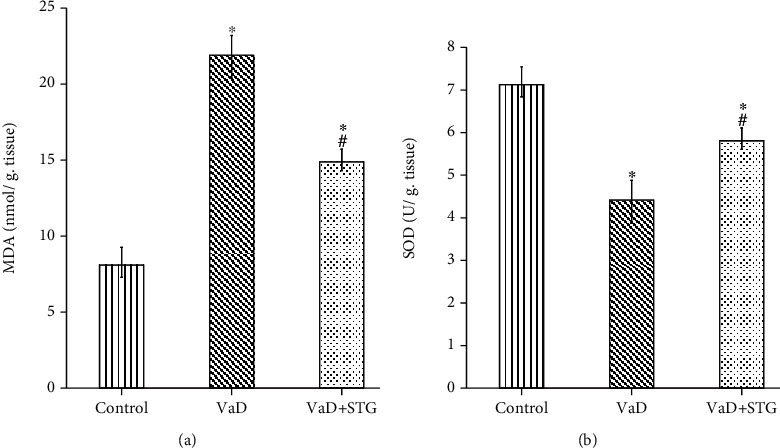
Effect of STG on in oxidative stress biomarkers in L-methionine-induced VaD (VaD: vascular dementia; VaD+STG: vascular dementia +sitagliptin; MDA: malondialdehyde; SOD: superoxide dismutase). Each group (*n* = 10) represents as mean ± SD. (a) The tissue MDA level. (b) The tissue SOD level. ^∗^*P* < 0.001 versus the control group; ^#^*P* < 0.001 versus the VaD group.

The tissue MDA level in the VaD group was significantly higher than that of the control group (22.01 ± 1.53 vs. 8.14 ± 0.89 nmol/g tissue, respectively, *P* < 0.001). Tissue MDA level in the VaD+STG group was significantly lower than that of the VaD group (14.8 ± 0.76 nmol/g tissue, *P* < 0.001). However, it was still significantly higher than that of the control group (*P* < 0.001) (see Figure 4(a)).

Moreover, there was a significant decrease in the SOD level in the VaD group compared with the control group (4.41 ± 0.35 vs. 7.07 ± 0.43 U/g tissue, respectively, *P* < 0.001). The SOD level in the VaD+STG group was significantly higher than that of the VaD group (5.75 ± 0.37 U/g tissue, *P* < 0.001), yet it was still significantly lower than that of the control group (*P* < 0.001) (see Figure 4(b)).

### 3.4. The Effects of STG on Inflammatory Biomarkers in L-Methionine-Induced VaD

**Figure 5:**
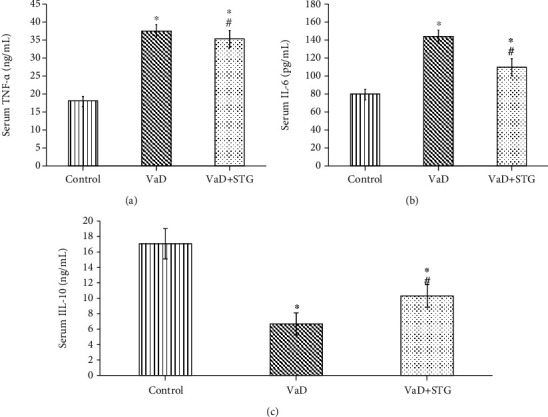
Effect of STG on in inflammatory biomarkers in L-methionine-induced VaD (VaD: vascular dementia; VaD+STG: vascular dementia +sitagliptin; TNF-*α*: tumor necrosis factor-*α*; IL-6: interleukin-6; IL-10: interleukin-10). Each group (*n* = 10) represents as mean ± SD. (a) The serum TNF-*α* level. (b) The serum IL-6 level. (c) The serum IL-10 level. ^∗^*P* < 0.001 versus the control group; ^#^*P* < 0.001 versus the VaD group.

The serum TNF-*α* level in the VaD group was significantly higher than that of the control group (37.23 ± 1.65 vs. 18.72 ± 0.89 ng/mL, respectively, *P* < 0.001). On contrary, the serum TNF-*α* level in the VaD + STG group was significantly lower than that of the VaD group (27.53 ± 2.14 ng/mL, *P* < 0.001), yet it was still significantly higher than the values of the control group (*P* < 0.001) (see Figure 5(a)).

There was a significant increase in the serum IL-6 level in the VaD group compared with the control group (147.33 ± 7.17 vs. 79.86 ± 3.74 Pg/mL, respectively, *P* < 0.001). Serum IL-6 level in the VaD+STG group was significantly lower than the values in the VaD group (110.15 ± 5.91 Pg/mL, *P* < 0.001); however, it was still significantly higher than that of the control group (*P* < 0.001) (see Figure 5(b)).

The IL-10 in the VaD group was significantly lower than that of the control group (6.67 ± 1.43 vs. 17.067 ± 1.96 ng/mL, respectively, *P* < 0.001). The IL-10 level in the VaD+STG group was significantly higher than the VaD group's (10.3 ± 1.48 ng/mL, *P* < 0.001), yet it was still significantly lower than that of the control group (*P* < 0.001) (see Figure 5(c)).

### 3.5. The Effects of STG on the Lipid Profile in L-Methionine-Induced VaD

**Figure 6:**
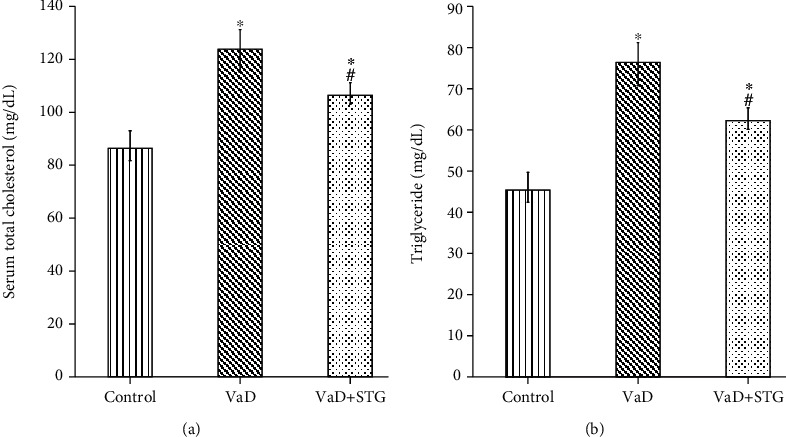
Effect of STG on lipid profile in L-methionine-induced VaD. (VaD: vascular dementia; VaD + STG: vascular dementia +sitagliptin). Each group (*n* = 10) represents as mean ± SD. (a) The serum total cholesterol level. (b) The serum triglyceride level. ^∗^*P* < 0.001 versus the control group; ^#^*P* < 0.001 versus the VaD group.

The serum total cholesterol level in the VaD group was significantly higher than that of the control group (124.5 ± 6.71 vs. 88.5 ± 6.05 mg/dL, respectively, *P* < 0.001). Moreover, the serum total cholesterol level in the VaD + STG group was significantly lower than that of the VaD group (107 ± 2.1, *P* < 0.001); however, it was still significantly higher than the control group (*P* < 0.001) (see Figure 6(a)).

A significant increase in the serum triglyceride level in the VaD group was present compared with the control group values (77.17 ± 3.12 vs. 47.33 ± 4.37 mg/dL, respectively, *P* < 0.001). The serum triglyceride level in the VaD + STG was significantly lower than that of the VaD group (62.8 ± 3.71 mg/dL, *P* < 0.001), albeit it remained significantly higher than that of the control group (*P* < 0.001) (see Figure 6(b)).

### 3.6. The Effect of STG on Hippocampal BDNF Levels in L-Methionine-Induced VaD

**Figure 7:**
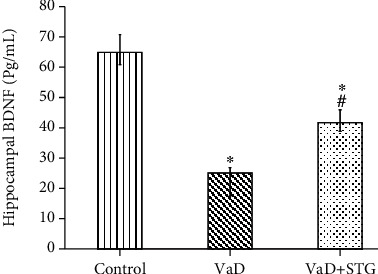
Effect of STG on in hippocampal BDNF level in L-methionine-induced VaD (VaD: vascular dementia; VaD + STG: vascular dementia +sitagliptin; BDNF: brain derived neurotrophic factor). Each group (*n* = 10) represents as mean ± SD. ^∗^*P* < 0.001 versus the control group; ^#^*P* < 0.001 versus the VaD group.

The mean value of hippocampal BDNF in the VaD group was significantly lower than that of the control group (25.85 ± 2.33 vs. 65.44 ± 2.62 Pg/mL, respectively, *P* < 0.001). Moreover, the hippocampal BDNF level in the VaD + STG was significantly higher than that of the VaD group (42.13 ± 2.93 Pg/mL, *P* < 0.001); however, it was still significantly lower than that of the control group (*P* < 0.001) (see Figure 7).

### 3.7. Histological and Immunohistochemical Evaluation

#### 3.7.1. Evaluation of the Thoracic Aorta

**Figure 8:**
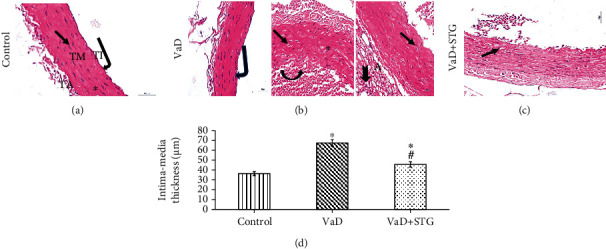
Representative photomicrographs of H&E-stained aortic sections of the different studied groups (×400, scale bar = 20 *μ*m) and measurement of the intima media thickness. (a) Control group: intact tunica intima (TI), normal endothelial lining (bent arrow), regularly arranged elastic lamina (asterisk), and normal smooth muscle cells (arrow) in between the elastic lamina in the tunica media (TM), tunica adventitia (TA). (b): VaD group: endothelial denudation (bent arrow), accumulated blood cells along the luminal aspect (arched arrow), irregularly arranged elastic lamina (asterisk), vacuolated smooth muscle cells (arrows), perivascular adipose tissue deposition (A), and inflammatory infiltrates within the tunica adventitia (notched arrow). (d): VaD + STG group: slight disruption in the tunica media (arrow). (d) Intima media thickness. Each group (*n* = 10) represents as mean ± SD. ^∗^*P* < 0.001 versus control group; ^#^*P* < 0.001 versus VaD group (VaD: vascular dementia; VaD + STG: vascular dementia +sitagliptin; H&E: haematoxylin and eosin).

Histological examination of the H&E–stained sections exhibited the normal structure of tunica intima, media, and adventitia in the control group (see Figure 8(a)). On the other hand, the VaD group showed endothelial denuding, accumulation of numerous blood cells in the luminal aspect of the aortic wall, extensive disrupted media with disorientation of the elastic lamina and vacuolation of some smooth muscle cells, and deposition of perivascular adipose tissue and inflammatory infiltrates in tunica adventitia (see Figures 8(b)). STG protected the aorta against the damaging effect of VaD. The VaD + STG group showed slight disruption of the media (see Figure 8(c)). Statistically, there was a significant increase in the aortic IMT in the VaD group compared with the control group (67.53 ± 3.40 vs. 36.31 ± 2.08 *μ*m, respectively, *P* < 0.001). On the contrary, the aortic IMT was significantly lowered in the VaD + STG group compared with the VaD group (45.65 ± 2.65, *P* < 0.001). However, it was significantly higher than the control group (*P* < 0.001) (see Figure 8(d)).

#### 3.7.2. Immunohistochemical Assessment of eNOS

**Figure 9:**
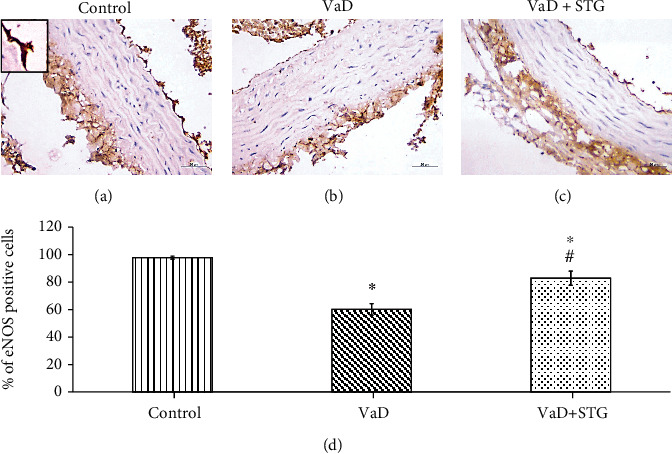
Representative photomicrographs of eNOS immunostaining of the aortic sections of the different studied groups (×400, scale bar = 20 *μ*m) and % of eNOS positive cells. (a) Control group, (b) VaD group, (c) VaD + STG group. VaD group showing downregulation of eNOS immunoreaction compared to the control group that relatively upregulated in the VaD + STG group compared to the VaD group. Inset: positive immunoreaction. (d) Percentage of the eNOS endothelial positive cells. Each group (*n* = 10) represents as mean ± SD. ^∗^*P* < 0.001 versus control group; ^#^*P* < 0.001 versus VaD group (VaD: vascular dementia; VaD + STG: vascular dementia +sitagliptin; eNOS: endothelial nitric oxide synthase).

Immunohistochemical assessment of the endothelial dysfunction revealed a significant decrease in the percentage of eNOS positive cells in the VaD group compared with the control group (60.20 ± 4.21 vs. 97.89 ± 1.11, respectively, *P* < 0.001). STG induced significant upregulation in eNOS immunoreaction in the VaD + STG group when compared with the VaD group (82.84 ± 5.24, *P* < 0.001). The eNOS expression was still significantly downregulated in the VaD + STG group than that of the control group value (*P* < 0.001) (see Figure 9).

#### 3.7.3. Evaluation of the Hippocampal CA1 Region

**Figure 10:**
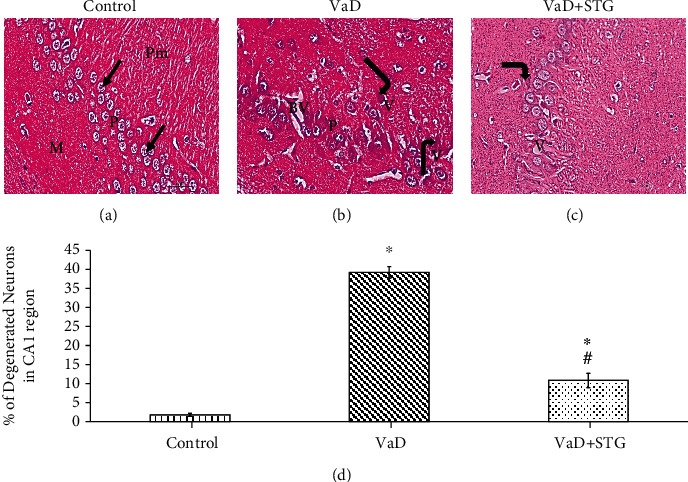
Representative photomicrographs of H&E-stained hippocampal CA1 sections of the different studied groups (×400, scale bar = 20 *μ*m) and % of degenerated neurons. (a) Control group: molecular (M), pyramidal (P), and polymorphic (Pm) layers. Compactly arranged pyramidal cells (arrows) are noted in the pyramidal layer. (b) VaD group: dispersed neurons in the pyramidal layer (P), deeply stained pyknotic nuclei (bent arrows) with perinuclear vacuolations (V), and dilated blood vessels (BV). (c) VaD + STG group: few pyknotic nuclei (bent arrow), slight vacuolation (V). (d) Percentage of degenerated neurons in the hippocampal CA1 region. Each group (*n* = 10) represents as mean ± SD. ^∗^*P* < 0.001 versus control group; ^#^*P* < 0.001 versus VaD group (VaD: vascular dementia; VaD + STG: vascular dementia +sitagliptin).

Histological assessment of the H&E-stained sections of the control group showed the normal structure of the hippocampus formed of molecular, pyramidal, and polymorphic layers (see Figure 10(a)). The VaD group showed many degenerative changes, in the form of dispersed neurons, deeply stained pyknotic nuclei with perineural vacuolation, and dilated blood vessels (see Figure 10(b)). On the other hand, the hippocampal CA1 region of the VaD + STG group revealed preservation of the normal structure, except for the slight neurodegenerative changes indicated by the appearance of vacuolation and few deeply stained pyknotic nuclei in the pyramidal layer (see Figure 10(c)).

Statistically, there was a highly significant increase in the percentage of degenerated neurons in the VaD group compared with the control group (38.85 ± 2.71 vs. 1.75 ± 0.20, respectively, *P* < 0.001). On the contrary, the percentage of the degenerated neurons was significantly decreased in the VaD + STG group compared with the VaD group (11.30 ± 1.72, *P* < 0.001); however, it was still higher than the control group (*P* < 0.001) (see Figure 10(d)).

#### 3.7.4. Immunohistochemical Assessment of the Caspase-3 Immunoreaction in the CA1 Hippocampal Region

**Figure 11:**
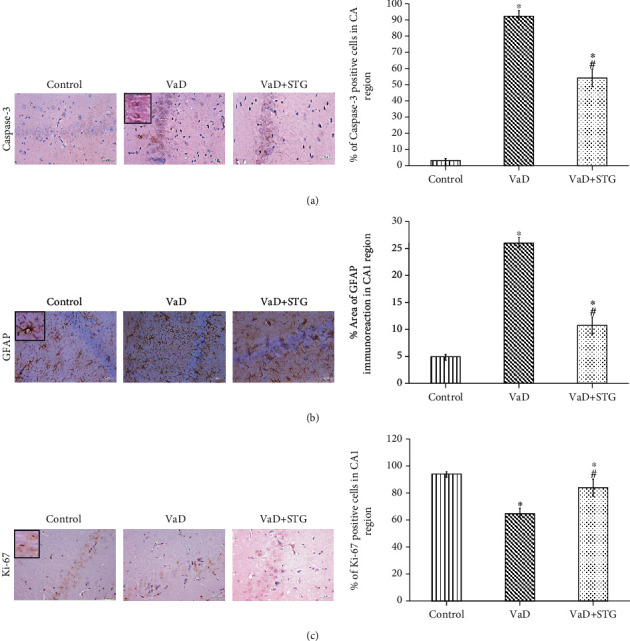
Representative photomicrographs of the hippocampal CA1 region immunostaining of the different studied groups (×400, scale bar = 20 *μ*m) and quantitative analysis of positive immunoreaction. (a) Caspase-3, (b): GFAP, (c): Ki-67. VaD group showing upregulation of caspase-3 and GFAP immunoreaction and downregulation of the Ki-67 immunoreaction compared to the control group. Downregulation of caspase-3 and GFAP and upregulation of Ki-67 are detected in VaD + STG group compared to VaD. Inset: positive immunoreaction. Each group (*n* = 10) represents as mean ± SD. ^∗^*P* < 0.001 versus control group; ^#^*P* < 0.001 versus VaD group (VaD: vascular dementia; VaD + STG: vascular dementia +sitagliptin; GFAP: glial fibrillary acidic protein).

There was a significant increase in the percentage of the caspase-3 positive cells in the pyramidal layer of the VaD group compared with the control group (91.73 ± 2.24 vs. 2.02 ± 0.28, respectively, *P* < 0.001). On the other hand, the VaD + STG group showed a significant caspase-3 immunoreaction downregulation compared with the VaD group (55.08 ± 2.66, *P* < 0.001). However, they it was higher than that of the control group (*P* < 0.001) (see Figure 11(a)).

#### 3.7.5. Immunohistochemical Assessment of the GFAP Immunoreaction in the CA1 Hippocampal Region

There was a significant increase in the area percentage of the GFAP immunoreaction in the VaD group compared with the control group values (26.07 ± 1.90 vs. 5.15 ± 1.00, respectively, *P* < 0.001). However, the GFAP immunoreaction was significantly downregulated in the VaD + STG group compared with the values of the VaD group (10.52 ± 1.61, *P* < 0.001). There was a significant difference between the control group and the VaD + STG group (*P* < 0.001) (see Figure 11(b)).

#### 3.7.6. Immunohistochemical Assessment of the Ki-67 Immunoreaction in the CA1 Hippocampal Region

The percentage of the Ki-67 positive cells in the pyramidal layer was significantly decreased in the VaD group compared with the control group (61.42 ± 3.35 vs. 97.47 ± 1.56, respectively, *P* < 0.001). Besides, there was a significant upregulation in the Ki-67 immunoreaction in the VaD + STG group compared with that of the VaD group (83.54 ± 2.66, *P* < 0.001). The Ki-67 immunoreaction was still significantly lower in the VaD + STG group than that of the control group (*P* < 0.001) (see Figure 11(c)).

## 4. Discussion

The incidence of VaD increases steeply, especially with age, adding to the growing epidemic of dementia worldwide [29]. In the present study, the potential neuroprotective role of STG in the L-methionine-induced VaD rat model was evaluated. To gain further insight into the potential underlying mechanisms, the beneficial effect of STG on oxidative stress, inflammatory markers, vascular structure, hippocampal apoptosis, gliosis, and neurogenesis were assessed.

In the present study, in the VaD group, where L-methionine induction occurred, cognitive deficit and endothelial dysfunction were revealed. The VaD group revealed a significant increase in serum Hcy levels. This agrees with previous study [30]. Hcy elevation was linked to changes in the structure and function of cerebral blood vessels, besides oxidative stress, both contribute to cerebral vascular dysfunction [31].

However, supplementation of VaD by STG showed a significant decrease in serum Hcy levels compared with the VaD group. To the best of our knowledge, this is the first study to elucidate the effect of STG in L-methionine-induced VaD.

In the current research, VaD significantly impaired cognitive performance. The open field test of the VaD group revealed an insignificant change of distance moved and decreased the number of rearing and time spent in the central zone when compared with the control group. This agrees with previous reported results [32]. Limiting these measures was found to represent increased anxiety in animals [33]. The EPM test results of the VaD group revealed decreased time in the open arms when compared with the results of the control group. Anxiety-like behavior is inversely proportional to the time an animal spent in the open arms. This agrees with previous study [34]. Thus, L-methionine induced a significant increase in the anxiety indicators in the open field and EPM tests.

Novel object recognition (NOR) of the VaD group revealed a lack of curiosity in investigating novel objects, indicating reduced recognition function and memory performance. This agrees with previous reported results [19]. During the Y-maze test, the VaD group showed a lower percentage of alternation. This agrees with previous study [35]. The altered behavior of rodents in the Y-maze suggested that spatial working or short-term memory was active [36]. The MWM test was used in the current study for the evaluation of spatial learning and memory. The VaD group's results demonstrated deterioration in the MWM test performance. This agrees with previous study [37], which concluded that L-methionine administration resulted in a considerable drop in MWM performance, indicating major impairment in memory acquisition and retrieval [38].

Treatment of VaD by STG showed a significant improvement of the cognitive deficit when compared with the VaD group. This was proved by the increased number of rearing and time spent in the central zone during the open field test and the increased time in the open arms of the EPM test, improving the anxiety parameters. Furthermore, the results of the VaD + STG group revealed an increased total time of exploration during the familiarization phase and an increased RI in the NOR and improved MWM performance. Also, there was an increased percentage of alternation during the Y-maze test. Thus, STG showed a major improvement in memory acquisition and retrieval. This concurs with previous study [39], which revealed that STG had a positive effect on memory function in Parkinsonian rats and improved performance in the MWM test. Also, these results agree with previous study [21], which revealed that STG had a positive effect on memory function in a rat model of aluminium-induced Alzheimer's disease through improved performance in the MWM test and NOR. This also concurs with previous study [40], which showed improved performance in the NOR test in high fat-fed mice. STG decreased anxiety in the EPM, agreeing with the results provided by previous reported results [41]. Overall, our findings suggest that STG could be a promising new candidate drug for protection against VaD in the future.

The increase in Hcy production in the VaD group resulted in the cognitive deficit. The pathophysiology of Hcy has been linked to increased oxidative stress in the hippocampus. The current study's findings back up this claim, with increased MDA, a lipid peroxidation marker, and a decrease in SOD concentration in the VaD group compared with the control group. This agrees with previous studies [9, 19]. Oxidative stress caused by excessive ROS production is a major contributor to tissue damage or an enhanced inflammatory response [42]. The VaD + STG group revealed a significant decrease in MDA level and increased SOD activity when compared with the VaD group. This agrees with previous studies [21, 43]. STG was proven to have strong antioxidant and free radical scavenging activities [43]. Moreover, Pintana et al. [44] demonstrated that STG attenuated brain oxidative stress and restored hippocampal mitochondrial function in rats. Indirect mechanisms for antioxidant activity of STG upregulated the expression of the nuclear factor erythroid 2-related factor 2 (Nrf2) [45]. Nrf2 is a key transcription factor involved in stress response to oxidative impairment and controlling the expression of antioxidants [46].

Neuroinflammation is characterized by the production of proinflammatory cytokines such as TNF-*α* and IL-6. It is often correlated to memory and cognition impairment [43]. There was a significant elevation of serums TNF-*α* and IL-6 and a significant decrease in IL-10 in the VaD group compared with the values of the control group. This agrees with previous reported results [19]. TNF-*α* triggers the inflammatory response by activating a cascade of cytokines, chemokines, and growth factors. As a result, it is classified as a proinflammatory cytokine [47]. IL-6 can be a trigger in nuclear factor kappa B (NF-*κ*B) signal transduction pathway promoting transcription and release of downstream inflammatory mediators, amplifying inflammatory responses [48]. IL-10 can suppress proinflammatory cytokine production [49]. Proinflammatory cytokines may cause the generation of ROS, which may cause an inflammatory response by activating the transcription factor NF-*κ*B. Then, NF-*κ*B is translocated into the nucleus and activates a number of inflammatory genes [50].

While the VaD + STG group revealed a significant decrease of TNF-*α* and IL-6 and a significant increase in the IL-10 when compared with the VaD group, This agrees with previous study [43], which revealed that STG was able to decrease the proinflammatory cytokines as STG has potent anti-inflammatory effects on both the nuclear and cytoplasmic levels [51]. DPP-4 inhibitors play a role in anti-inflammation by influencing the innate immune system, such as monocyte/macrophage activation, T cell activation, and inflammatory factor production in vivo [52]. Hu et al. [53] found that STG therapy might decrease NF-*κ*B activation as well as cytokine expression. The activation of the NF-*κ*B pathway increases transcription and the release of cytokines such as IL-6, IL-1, and TNF- *α* [54].

The detected cognitive deficits in the VaD group were mostly attributed to the impact of the vasculopathy with the upcoming neurodegenerative changes affecting CA1 hippocampal neurons. The HHcy-induced vascular dysfunction was proved in the current work by the significant increase in total cholesterol and triglyceride levels in the VaD group as compared with the control one. This agrees with previous studies [30, 37], besides the concomitant histopathological alteration of the aorta. The observed histological alterations were nearly like that recorded by previous study [35]. Significant downregulation of eNOS confirmed the vascular dysfunction. Aorta was examined as a representative vessel to assess the vascular dysfunction. The endothelial changes in the aorta and increased IMT reflected atherosclerotic changes as postulated by previous study [55]. Moreover, Othman et al. [56] clarified that thickening aortic wall and distorted elastic lamellae might be the factors that contributed to the impairment of the structural integrity of the aorta.

Normally, nitric oxide (NO) is primarily generated by eNOS in the vascular endothelial layer and plays a crucial role in the regulation of vascular tone and blood flow [57]. So, the recorded significant eNOS downregulation in the VaD group compared with the control, in the present work, could reflect the endothelial dysfunction. This agrees with previous reported results [58]. Bhatia and Singh [59] declared that the induction of HHcy decreased the activity of eNOS. Hemanth Kumar et al. [35] attributed this to the formation of S-nitroso-Hcy following the exposure of endothelial cells to Hcy with a subsequent decrease in the bioactivity of NO. Furthermore, previous studies attributed the vascular dysfunction to the increased lipid concentration besides the initiation of inflammatory processes [56, 60]. The reported accumulated blood cells and perivascular adipose tissue deposition indicated the presence of active inflammation as clarified by previous study [61].

Sitagliptin (STG) exhibited a considerable reduction in serum total cholesterol and triglyceride levels when compared with the VaD group. This agrees with previous reported results [20]. In the present study, STG improved the histopathological picture with a concomitant significant increase of eNOS immunoreaction compared with the nontreated VaD group. This concurs with previous study [62], which detected the hypolipidemic effect of STG. Liu et al. [63] referred the improvement of the vascular dysfunction in a hypertensive rat model to the upregulation of GLP-1 that restored the NO bioavailability and increased the eNOS activity.

The hippocampal CA1 region is sensitive to ischemia [64]. The dilated blood vessels observed in the hippocampal CA1 region in the VaD group could reflect the occurrence of transient ischemic attacks with subsequent neurodegenerative changes as clarified by previous study [65]. The recorded neurodegenerative changes were indicated by the presence of pyknotic nuclei with perinuclear vacuolation and the significant increase in the percentage of degenerated neurons in the VaD group. This concurs with previous studies [59, 64]. This was mostly attributed to the increase in apoptotic cell death that was confirmed in the current study by the significant upregulation in the caspase-3 immunoreaction. This also agrees with previous study [8], which detected the implication of the apoptosis signaling in the hippocampus of the VaD model. Kizilay et al. [66] attributed the initiation of apoptosis pathways to the existence of oxidative stress. However, STG supplementation resulted in a significant reduction of the percentage of degenerated neurons compared with the VaD group values. This could be attributed to its antiapoptotic effect as indicated by the significant downregulation of caspase 3. This agrees with previous study which declared that STG decreased caspase-3 expression in the brain tissues following ischemia/reperfusion injury [18]. Kizilay et al. [66] attributed the antiapoptotic role of STG to its antioxidant property and its ability in regulating calcium release from the endoplasmic reticulum (ER), hence, protecting against ER stress.

There was a significant upregulation in astrocyte marker, GFAP, immunoreaction in the hippocampus of the VaD group, reflecting the implication of gliosis in the VaD pathogenesis. This agrees with previous reported results [67]. Baydas et al. [68] attributed that to HHcy-induced glial cell sensitization. Moreover, previous studies clarified that increased tissue GFAP immunoreactivity was a sensitive indicator of neuronal injury and neuroinflammation [69, 70]. However, the VaD + STG group results revealed significant downregulation of GFAP immunoreaction when compared with the VaD group. This concurs with previous reported results [71], who noted that STG significantly reduced GFAP in a febrile seizures rat model. They attributed that to its anti-inflammatory properties.

There is growing evidence that adult neurogenesis is critical for the brain function, and it is impaired by ROS overload [72] and neuroinflammation [69]. BDNF has been linked to synaptic plasticity, learning, memory capability [73], and neurogenesis [74].

To the best of our knowledge, no research was conducted on the effect of STG-induced hippocampal neurogenesis in VaD. As a result, the effect of enhanced hippocampus neurogenesis generated by STG on cognitive impairment induced by L-methionine in rats was validated. The VaD group revealed impaired neurogenesis via a significant decrease in BDNF and Ki-67 immunoreaction compared with the results of the control group. This concurs with previous reported results [75], which found that prolonged cerebral hypoperfusion decreased BDNF expression and with previous study [76] in which BDNF levels decreased in the VaD model induced by common carotid ligation. The decreased BDNF level in VaD group resulted in impaired cognitive performance. BDNF has been identified to influence cognitive processes [12]. The downregulation of Ki-67 expression could be attributed to the decreased BDNF level [77].

Sitagliptin (STG) administration significantly ameliorated the hippocampal neurogenesis and neuronal plasticity through the significant increase in the hippocampal BDNF level and upregulation of Ki-67 immunoreaction in the hippocampal neurons compared with the VaD outcomes. This concurs with previous study [78], which noted that DPP-4 inhibitor treatment in nondiabetic rats elevated the BDNF in the cortex and the whole forebrain. Also, STG improved memory deficits in Parkinson's disease rats via upregulation of the BDNF expression [39]. The role of STG in neurogenesis could be attributed to its antioxidant and anti-inflammatory properties. In addition, Bachor et al. [79] attributed the neurotrophic effect of STG to its role in protecting the proliferation of neural progenitor cells.

Overall, our findings suggest that STG could be a promising new candidate drug protecting against VaD in high-risk people in the future. This beneficial effect was mediated by STG's ability to attenuate oxidative stress, inflammation, vascular alterations, neuronal apoptosis, and gliosis, in addition to its neurotrophic effect via increased hippocampal BDNF level.

## 5. Conclusions

Finally, the incidence of VaD has recently been shown to be growing, even though none of the available medicines yield a significant reduction in disease progression. To halt the progression of the disease, a considerable investment in medical research is required to discover novel drugs with high clinical efficacy that target the underlying pathogenic pathways. In rats with L-methionine-induced VaD, STG did indeed ameliorate cognitive deficits. It had neuroprotective effects via its antioxidant, anti-inflammatory, and antiapoptotic properties. Besides, it increased BDNF levels in rats in the current investigation, suggesting that it could help halt disease development.

## Figures and Tables

**Table 1:** Effect of sitagliptin on percentage of change of latency between day 1 and day 5 in MWM test in L-methionine-induced VaD.

Group parameter	Control group	VaD group	VaD+STG group
Percentage of change of latency between day 1 and day 5	−67.88 ± 3.28	−29.18 ± 1.56^∗^	−49.20 ± 2.20^∗^^#^

MWM: Morris water maze; VaD: vascular dementia; VaD+STG: vascular dementia +sitagliptin. Each group (*n* = 10) represents as mean ± SD. ^∗^*P* < 0.001 versus the control group; ^#^*P* < 0.001 versus the VaD group.

## Data Availability

The data that support the findings of this study are available from the corresponding author upon reasonable request.
